# Isolation in COVID, and COVID in Isolation—Exacerbated Shortfalls in Provision for Women's Health and Well-Being Among Marginalized Urban Communities in India

**DOI:** 10.3389/fgwh.2021.769292

**Published:** 2022-01-04

**Authors:** Lakshmi K. Josyula, Shrutika Murthy, Himabindu Karampudi, Surekha Garimella

**Affiliations:** ^1^The George Institute for Global Health, Hyderabad, India; ^2^Prasanna School of Public Health, Manipal Academy of Higher Education, Manipal, India; ^3^University of New South Wales, Sydney, NSW, Australia; ^4^The George Institute for Global Health, New Delhi, India; ^5^Dalit Bahujan Resource Centre, Guntur, India; ^6^Institute of Public Health, Bengaluru, India

**Keywords:** lived experiences, women waste pickers, COVID-19, health and well-being, marginalized urban communities, India

## Abstract

This paper describes the lived experiences of health seeking, health care recourse, and well-being of women waste pickers, a highly marginalized sub-population in urban areas in India, highlighting the intersectionality of gender, socioeconomic and cultural contexts, and occupational hazards that they face, as studied by a research team engaged in participatory action research with waste workers in urban India. We note the impact of the superimposition of the COVID-19 pandemic, with the restrictions on movement and access to livelihoods, social support, and health care, and policies made and enforced in a fragmented manner, on the already deprived conditions of the waste pickers. We reflect on the women waste pickers' practices of health seeking, their access to health care, the provisions made for them and made use of by them, and the support they could tap in protecting and restoring their health. A range of these experiences is illustrated through three case studies. Finally, recommendations are made for better provision for women's health and well-being, and improved preparedness for emergency situations.

## Introduction

Working with waste in urban areas in India comprises a wide range of occupations, from street cleaning, to door-to-door garbage collection, to picking, sorting, sale, and processing of reusable and recyclable waste from streets and dumps (1); and various degrees of formality of employment, from regular government jobs, to fixed-term contracts with the government or private organizations, to informal self-employment. Waste workers generally belong to marginalized urban communities facing multiple social, economic, and occupational disadvantages (2, 3).

Waste workers' experiences of seeking and practising livelihoods, protecting and restoring health, and interacting with community members evince intersectionality of gender, caste, religion, region of origin and migration status, language, and nature of employment (regular, contractual, informal, or self-employed) (3). The challenges posed by the COVID-19 pandemic have been superimposed on the pre-existing privations faced by waste picker communities. In particular, the experiences of, and challenges faced by, women in the waste picking communities, which demonstrate the concurrent and compounded impacts of gender, socioeconomic status, cultural identity, and occupation, have tended to be grouped without adequate disaggregation with the experiences of all waste workers, or worse, all socioeconomically disadvantaged urban communities.

## What We Did

Accountability for Informal Urban Equity (ARISE) (4) is a consortium of interdisciplinary research hubs across Africa, South Asia, and the United Kingdom, working on addressing the challenges of ill-health, inequity, and insecurity in informal urban settlements in low- and middle-income countries (LMICs), through participatory research and community action to build government accountability, and inform policy change. At the George Institute for Global Health, India, one of the ARISE hubs, we focus our work on waste workers across three states in India, through partnerships with civil society organizations working with the communities in these action sites.

Over the course of our work on the ARISE project, we conducted a review of the literature on waste workers, with an emphasis on waste pickers in the informal sector. We engaged in participant and non-participant observation, interviews, focus group discussions, community meetings, and workshops with waste picker communities, and civil society organizations, to understand the lived experiences of waste pickers, and the physical, social, and policy environments that they live and work in. We undertook a detailed policy landscaping exercise, including key informant interviews, to review policies related to the health, health care recourse, occupational health and safety, and social security of waste workers. Further, we examined 97 COVID-19 specific policies made by the Indian central and state governments, and analyzed policies that pertained to the health, security, livelihood, and well-being of disadvantaged urban communities.

## What We Found

Preliminary learnings from our review of the literature highlight waste workers' position among the most disenfranchised, marginalized, and oppressed communities in India (5). Women waste workers, in particular, experience excessive discrimination and precarity, shouldering multiple burdens arising from gendered division of labor and informal work arrangements, having inadequate access to sexual and reproductive health opportunities and care, and being invisible in existing leadership spaces (6–9).

While there are no clear estimates on how many women are involved in waste work, the information that is available from some cities indicates that women constitute up to 70–90% of this workforce (10). Low literacy levels and pre-existing gender differentials make occupational mobility for women waste workers more difficult than for men (11, 12). Even within the waste-to-recovery chain, positions of authority are predominantly occupied by men (13–15). Women waste workers in India tend to perform the most physically arduous tasks, are predominantly engaged in informal work contracts or are own account workers, and get trapped in exploitative arrangements (11). Consequently, they are often not recognized as “workers,” and are thereby excluded from attaining and accessing social security and welfare schemes. These vulnerabilities of women waste workers are insufficiently examined in scholarship on gender, labor, informal work, the waste economy, and health systems. Research on the health and well-being of women waste workers is mostly limited to a few discrete studies on occupational health and safety (16–18). There is a dearth of relational, intersectional, and contextual research that explores and understands the health and well-being concerns and embodied experiences of women waste workers.

In the advent of the COVID-19 pandemic, the federal and state governments in India developed a slew of orders, guidelines, and rules to deal with the disease and its impact (19). Issues specific to COVID-19 that found mention in policies were containment, including lockdowns; testing; stranded persons, particularly migrants, students, and pilgrims; waste management, especially disposal; discrimination, and inhumane treatment of persons in the pandemic; and specific groups of persons, e.g., senior citizens, vulnerable to COVID-19 infection and complications. However, most of the social assistance schemes were directed at population groups that were broadly categorized as “poor” and “vulnerable.” The majority of the policies did not apply to the specific precarities and vulnerabilities faced by waste workers in the informal sector, but were aimed at assuring food and shelter to those who had lost their livelihoods in the pandemic. This included schemes for the provision of relief kits and meals, but these too were limited to a 3-month period at the beginning of the lockdown in 2020. Most of the policy announcements were built on to pre-existing schemes (see Supplementary Material 1 for a list of policies pertaining to waste workers) and did not adequately address the impact of loss of work, hunger, and inability to access health care.

Some state governments in India provided financial assistance to all frontline workers, including sanitation workers, who were involved in the efforts against COVID-19, and thereby had disproportionate exposure to the disease. Among waste workers, only tenured or regular sanitation workers engaged in the cleanliness and upkeep of cities had policies specifically addressing their health insurance, financial incentives, and personal protective equipment (PPE) requirements. We noted no reference or provision for waste workers in informal employment, particularly migrant workers who lacked government-issued documentation. While many of the policies developed and enforced to address the transmission of COVID-19 were sound in biological and epidemiological terms, they failed to take into account the lives, and especially the constraints, faced by disadvantaged communities, leading to situations in which people found themselves protected to some extent from COVID-19, but vulnerable to loss of livelihoods, precarious nutritional intake, disrupted social interaction and support, and violence.

“*If the NGO had not helped us with relief packages, we would have died of starvation, not corona.”—*informal waste picker, Andhra Pradesh.

### Pre- and Intra-Pandemic Experiences of Health Recourse

The pre-COVID-19 scenario of women waste pickers' health seeking, provision, and access was one of lack of awareness of services, such as antenatal check-ups, nutrition supplementation, vaccination, maternity/sick leave; hesitation to access health protection, screening, and curative services; difficulty in accessing health care owing to conflicting occupational and family priorities, administrative requirements, and pragmatic needs, such as of transport; and adverse experiences of poor quality care and hostility in interactions with the health system. Support from civil society organization champions as well as community health volunteers was acknowledged as essential to shepherd women through the health system. Some families had availed themselves of the provisions offered by governments for the care of children in government hostels, and for education and nutritional supplementation for community-dwelling children, while many families were unable to access these provisions owing to administrative hurdles that they could not overcome.

In the course of the pandemic, restrictions applied to prevent and contain COVID-19 impeded women's lives, livelihoods, and their families' health and well-being. With the lockdowns imposed early in the pandemic, children from disadvantaged communities had to return home from government hostels which supported their boarding, lodging, and educational expenses. The switch of residence from hostels to parents' homes meant that all expenses on the children switched abruptly to the unprepared parents, whose livelihoods had been stalled at the same time. Relief measures proposed and implemented by government, civil society organizations, and individual philanthropists were helpful, but piecemeal, and did not reach all in need. Households were compelled to incur loans, and experienced further compromised nutrition, and further impaired access to information. COVID-19 screening and treatment were associated with fear, misinformation, stigma, and adverse impact on the care of family members. Routine or emergent non-COVID-19 healthcare were affected in these communities, with health care facilities out of bounds, health care providers under pressure, and transport exorbitantly priced. The burden of care as well as the brunt of the distress and vulnerability to violence within the home fell to women.

### Health Problems Frequently Encountered by Women Waste Pickers

Most waste pickers in the informal sector cannot afford protective gear, making them vulnerable to mechanical, chemical, and biological injuries. Considering that activities that involve direct contact with mixed waste are predominantly carried out by women and that too by informal workers, they are the persons who need PPE the most but are least likely to have and use them. The few waste workers who do use some protective equipment are generally provided them by their employers.

Most women report acute or chronic pain in one or more parts of their body, including the head, eyes, arms, stomach, pelvic region, knees, and legs. Women often neglect such pain and sickness until they cannot function without treatment, at which point they take recourse to local pharmacies, private or government clinics, or local health traditions and home remedies. Some women, despite having health cards issued by the state government, are disinclined toward seeking care at government health care facilities on account of the waiting periods and adverse interactions involved at these institutions.

Women waste pickers have practically no access to civic amenities such as toilets while they are working. They are forced to constrain their toilet use to before and after work timings, a situation that is especially inconvenient during menstruation. Menstrual hygiene management is a highly neglected aspect of waste pickers' health, with shortfalls in awareness, affordability, safe storage, and amenities for use, changing, post-use treatment of reusable menstrual absorbents, and disposal of one-time use products.

Gynecological morbidities leading to repeated sickness, chronic anemia, and impaired functioning, and paving the way to surgeries such as hysterectomy, are common. Antenatal check-ups and institutional deliveries are not the norm among waste picker women. Pregnant women work until a few days before childbirth, and return to work within about a month after childbirth, taking the infant with them or leaving the infant at home with a caretaker, if any. Longer periods of post-partum rest at home represent lost income, and are not sought.

Waste pickers experience constant insecurity during work, as they perceive that residents suspect them of thieving. Many waste pickers report having to check in at the local police station every week in the course of police cases lodged against them by residents. During the pandemic, waste pickers observe that they are shunned as potential disease-transmitters by residents of the areas that they work in, making it additionally difficult for them to obtain work. Women in waste picker communities, besides experiencing several forms of social and structural violence and neglect, are also exposed to domestic violence perpetrated by drunk men.

### Case Studies

We illustrate some of these findings through case studies of women waste pickers' experiences in health management and healthcare recourse, from two states in India. Topics explored in our discussions and observations included: the current intra-COVID-19 situation of family and social support, engagement with civil society organizations, and awareness of government health services; how the intra-COVID-19 situation differed from the pre-COVID-19 one in terms of policy, implementation, provision, and financial and social support; the impact on work and income; and needs—met and unmet—related to services, amenities, security, and equity.

Case study 1: Shivani^*^, 44, Andhra Pradesh

Shivani was married at the age of 15, and had three children in quick succession. She has always been the primary breadwinner of the family. Her alcoholic husband not only did not contribute to the household, but spent a large proportion of her earnings on alcohol besides. In her mid-twenties, she suffered painful and heavy menstruation, which she tried to keep at bay with over-the-counter painkillers as she continued to work despite her pain and discomfort. She put off check-ups and hospital visits to save time and money, until her condition became too serious to ignore. Finally, she visited a hospital where a severe uterine infection was diagnosed, and the doctor expressed the potential risk of cancer if the problem continued unchecked. She underwent hysterectomy in a private hospital at the age of 26. She received moral and financial support from her parents through this crisis. Post-hysterectomy, Shivani is relieved that she is enabled to work all month without any constraints posed by menstruation.

Three years ago, she had typhoid and fell unconscious while working. She got admitted to a government hospital where she underwent multiple tests, and was diagnosed with diabetes. While the consultation was of free of cost, she had to bear the medication expenses, although medications are notionally to be made available free of cost too (20). Shivani found herself in the quandary of taking medication to control blood sugar levels, but not having enough food to eat commensurate with the medication. Over time, she developed low blood pressure and had seizures at times while at work. She cultivated a routine of regular check-ups at the government clinic, and replenishing her medications at a private pharmacy (21). Her deteriorating health left her capable of work only about half the month.

The lockdowns occasioned by the COVID-19 pandemic placed critical constraints on Shivani's livelihood and slashed her income drastically as access to waste picking was not allowed in many parts of the city. In parallel, as a measure to tackle the pandemic, (formally employed) municipal workers were instructed to step up their waste collection activities, leaving practically no scope for informal waste pickers to work. An additional obstacle that waste pickers faced was the hostility that residents expressed, viewing waste pickers as potential disease-transmitters.

With the reduction in income, the entire family's nutrition worsened. Even the infrequent relief packages from the government, NGOs, and individual philanthropists could not fulfill all the household's needs. The lockdowns affected Shivani's healthcare routine adversely as well: the government hospital did not allow patients inside the facility, and the patients did not feel as well-treated by the doctors as they had in pre-COVID-19 consultations. The regularity of Shivani's tests for diabetes was affected, with the interval between tests prolonged to 2–3 months.

In the past 2 months, Shivani has had a further health setback: An inch-long nail from the waste she was picking through, pierced her foot and led to a non-healing wound, which comes in the way of her walking, and therefore working. Besides the impact of this on the fulfillment of her immediate needs, Shivani is apprehensive about its implications for her ability to repay the debts that she incurred in the past few years for the celebration of her daughters' weddings. She reports that her sleep is disturbed nightly with these worries, and that her peace of mind is lost. She seeks support for an alternative livelihood, such as vegetable vending, which would not require her to walk a lot, to help her get back on track with earning, and ideally, saving.

Case study 2: Saroj^*^, 24, Himachal Pradesh

Saroj was born in Shimla, into a migrant family of manual laborers from Nepal. In childhood, her family kept traveling between Nepal and Shimla, as her parents' work assignments dictated. The continual shifting affected the continuity of her education, which she, although a good student, had to give up when she was around 12 years of age to take up manual labor to contribute to the household income.

Saroj got married at the age of 14 years, and was pregnant with her first child at 16, although she lied about her age for fear of admonishment from health care providers for marrying and getting pregnant so young. Based on this encounter with the health system, her Aadhar card (an identity card issued by the government of India) reflects an older age.

All three of her children were delivered in government hospitals, however, her experiences of care were not uniform. Despite undergoing routine ante-natal check-ups at the government hospital, Saroj was not aware of the requirement of an HIV test prior to admission for labor. She was denied admission to a government hospital for women and children on grounds of not having been tested for HIV. She was rushed by her family to another government hospital for delivery as she was already in labor and needed care urgently. The requirements for admission differed between these government hospitals.

After her third delivery, Saroj suffered a degree of uterine prolapse, for which she sought the help of a local masseuse, who administered a brief massage to adjust the position of her uterus. Saroj, who was engaged in manual labor, found that lifting and carrying loads aggravated her uterine prolapse. She switched to office cleaning, a job that did not involve lifting and carrying loads. However, the long working hours came in the way of childcare, especially since she was the only adult in the household on a regular basis, as her husband lives and works at an orchard around 2 hours away. Saroj then switched to door-to-door garbage collection, which has more convenient work timings. However, this job involves lifting and carrying loads across hilly terrain, aggravating her uterine prolapse, and necessitating frequent visits to the masseuse.

Both Saroj and her husband contracted COVID-19 in the second wave of the pandemic. Saroj had had a fever and cough for about a week, through which she continued to work to ensure uninterrupted income, before the death of one of her neighbors prompted the local health workers to set up a testing camp in their locality. On testing, Saroj and her husband were found to be COVID-positive. The local Primary Health Centre as well as the community health volunteer coordinated their care at this time, delivering medication and facilitating tests. The local government doctor and community health volunteer also coordinated continued support of the COVID-positive community members through a WhatsApp group for follow-up and query resolution. Saroj had certain incorrect beliefs about the course of COVID-19, such as that perspiration caused by ambient heat would eliminate the disease. Saroj's family of five had to undertake isolation as a unit, although the children were not infected, as they did not have the resources to quarantine the children elsewhere. Their living arrangements are such that although each household has access to a separate toilet and bathroom, the access is through a common corridor. So, the family had to exert constant caution to isolate from neighbors. Saroj and her husband had a harrowing time dealing with their own sickness, and their worries about the children's susceptibility to COVID-19 as well as their future vulnerability in the event of the loss of their parents from COVID-19.

Saroj's children were going to school before the pandemic. Online classes were not a feasible and sustainable option for Saroj. She enrolled her children for private tutoring with a local teacher, but is distressed to note that the teacher is very harsh about punishment for schoolwork not done as specified. However, Saroj has no other affordable option for her children's education at this time.

Besides her gynecological troubles, Saroj suffers frequent headaches, for which she uses over-the-counter painkillers or home remedies of spiced oil. She also experiences distress in encounters with some residents in her work zones, who sometimes make discriminatory remarks about her occupation of garbage collection, which sting her. Saroj derives a sense of belonging and social support in her community that has a shared history of migration from Nepal, and tends to be isolated from other communities.

Case study 3: Srilatha^*^, 30, Andhra Pradesh

Srilatha lives in a makeshift settlement a few meters from a dump yard. After an early marriage in her teens, she had two daughters, whom she gave birth to at home. She reports that her pregnancies and childbirth were uneventful, and that her children were healthy. Three years ago, her husband took their daughters away and deserted her. She migrated to the settlement next to the dump yard then, and has had no contact with her husband or her children since. Srilatha heard recently that her younger daughter is dead, however, she does not know the circumstances of her death.

Two years ago, Srilatha began living with a 42-year old man who had been abandoned by his wife. She became pregnant during the COVID-19 pandemic. She did not have any routine antenatal check-up, and was not eligible for any government-sponsored benefits at the Anganwadi center (for supplemental nutrition) for want of documentation. Srilatha had made no plans for an institutional delivery. However, she unexpectedly went into labor in the 7th month of pregnancy, and got admitted to a government hospital with the assistance of NGO volunteers. Although she underwent normal labor and delivery, the baby was stillborn. The doctors explained that the baby had died in the womb from an infection. Srilatha attributes the death of her unborn child to her “negligence,” which is how she describes her regimen of low priority for cleanliness, nutrition, and rest, and steady routine of waste picking throughout her pregnancy. She rues the low level of personal and environmental hygiene that she can reasonably maintain in waste picking and living next to a dump yard, with no access to civic amenities.

To compound Srilatha's distress, the nurses at the hospital refused to hand over the body of her stillborn child to her without receiving some payment, as bribe. The intervention of the NGO volunteer, who was not unused to the demand for bribe for the handover of babies to mothers at the hospital, ensured that Srilatha got her baby's body without having to expend money for it.

Srilatha declares that she is not bothered about contracting COVID-19 or any other disease. She is completely against the formal healthcare system itself, and believes that hospitals are unsafe and unaffordable. She prefers local health traditions to address any sickness she suffers, and to give birth at home rather than in hospital. Had the NGO volunteers not been available to assist her, she would have given birth at home again rather than undergo the difficult process of hospital admission without government-issued documents, and have to incur transport and other expenses as well.

Srilatha considers herself tough and resilient, mentally and physically. She believes that this hardiness is critical to her daily toil to support herself and her family. She recounted her return to waste picking in a week after her delivery and child's death in the womb, as “nothing should stop [her] from working.”

## Discussion

Women waste pickers demonstrate intersectionality of multiple disadvantages in their personal, social, and occupational lives. Their deprived educational and sociocultural backgrounds predispose them to low health awareness, and their low autonomy leaves them with very little agency and behavioral control in the face of high vulnerability and varied responsibilities.

The health and well-being needs of women waste pickers are not understood and addressed adequately. They are often clubbed with the occupational pitfalls common to men and women, without regard to the differential experiences based on sex and gender. This is particularly problematic considering that the genders are non-uniformly divided among the range of activities in waste work. Certain activities, such as picking recyclable waste on the streets and in dump yards, are disproportionately performed by women, and their execution needs to take into account the other health and civic conditions of women, rather than be considered as a neutral human response to exposure to particular physical, chemical, or biological hazards.

Health and well-being are an early casualty in emergency situations affecting entire populations, such as the COVID-19 pandemic, but also in the frequent and varied crises endured by communities and individuals experiencing multiple disadvantages and living and working in precarious conditions. Economic challenges clearly move the health and well-being of all household members down the list of priorities. Further, the lowest priority is accorded to the health and well-being of girls and women, often by themselves too. The pervasive sociocultural norms prioritizing men, particularly those engaged in earning for the household, persist in the communities of waste workers notwithstanding the frequent occurrence of unemployment in males, and the disproportionate burden of economic, social, and nurturing support falling on the female household members.

### Recommendations

Our pre- and intra-pandemic studies highlight the imperative to assure the following basic facilities for the health and well-being of waste picker (and other) women:

- civic amenities, including secure housing, electricity, water, and sanitation facilities;
- livelihood security, through registration, recognition of waste work, and assurance of wages;
- access to reproductive and child health, including menstrual hygiene management, antenatal care, institutional delivery, immunization, and nutrition;
- education;
- health care, encompassing health protection, disease and injury prevention, health promotion, and therapeutic options;
- occupational safety, including the provision and enforcement of PPE use, and regular check-ups for secondary prevention of occupational disorders;
- security from crime, and intentional injuries; and
- opportunities for social participation.

In addition, societies need to recognize the vital contributions of waste workers in ensuring the cleanliness of urban areas and environmental sustainability. In a context of such institutionalized provision and societal sensitization, responding effectively and equitably to a crisis such as a disease outbreak or natural disaster would not deprive disadvantaged girls and women of essential health access as the response to the COVID-19 pandemic has.

## Data Availability Statement

The raw data supporting the conclusions of this article will be made available by the authors, without undue reservation.

## Ethics Statement

Ethics clearance for this work was obtained from the Liverpool School of Tropical Medicine Research Governance and Ethics Office, and the Institutional Ethics Committee, The George Institute for Global Health, India. Participants provided written informed consent for participation in this study.

## Author Contributions

LJ drafted and revised the manuscript. SM contributed to a case study and to manuscript revision. HK contributed to two case studies. SG guided the development of the manuscript. All authors engaged in data-collection, analysis, and interpretation of findings.

## Funding

The GCRF Accountability for Informal Urban Equity Hub (ARISE) is a UKRI Collective Fund award with award reference ES/S00811X/1.

## Conflict of Interest

The authors declare that the research was conducted in the absence of any commercial or financial relationships that could be construed as a potential conflict of interest.

## Publisher's Note

All claims expressed in this article are solely those of the authors and do not necessarily represent those of their affiliated organizations, or those of the publisher, the editors and the reviewers. Any product that may be evaluated in this article, or claim that may be made by its manufacturer, is not guaranteed or endorsed by the publisher.

## Footnotes

^*^Names changed to preserve anonymity.
