# Supplementary material for: Isolation in COVID, and COVID in Isolation—Exacerbated Shortfalls in Provision for Women's Health and Well-Being Among Marginalized Urban Communities in India
Source: Front Glob Womens Health. 2022 Jan 4;2:769292. doi: 10.3389/fgwh.2021.769292 (PMC8764266; doi:10.3389/fgwh.2021.769292)
Supplement: Supplementary file 1 [file Data_Sheet_1.PDF]

Supplementary File 1

Local, state, national, and global policies pertaining to the health of all persons, workers in general, and waste workers in particular, reviewed in the ARISE project

1. Government of India, **The Workmen's Compensation Act, 1923** (Ministry of Labour and Employment ed., 1923).
2. United Nations, **United Nations Universal Declaration of Human Rights, 1948**.
3. Government of India, **Maternity Benefit Act, 1961** (Ministry of Labour and Employment ed., No. 53 of 1961 ed. 1961).
4. Government of India, **Bajaj Committee Report** (Ministry of Health and Family Welfare ed., 1986).
5. Government of India, **Indira Gandhi National Old Age Pension Scheme, 1995** (Ministry of Rural Development ed., 1995).
6. Government of India, **National Family Benefit Scheme, 1995** (Ministry of Rural Development ed., 1995).
7. Government of India, **Report of the Second National Commission on Labour, 2002** (Ministry of Labour and Employment ed., 2002).
8. Government of India, **National Rural Health Mission: Framework for Implementation** (Ministry of Health and Family Welfare ed., 2005).
9. Government of India, **Janani Suraksha Yojana (JSY), 2005** (Ministry of Health and Family Welfare ed., 2005).
10. Government of India, **Report of Performance Audit on "Management of Waste in India"** (Comptroller and Auditor General of India ed., 2007).
11. Government of India, **Report on Conditions of Work and Promotion of Livelihoods in the Unorganised Sector** (National Commission for Enterprises in the Unorganised Sector ed., 2007).
12. Government of India, **Rashtriya Swasthya Bima Yojana, 2008** (Ministry of Labour and Employment ed., 2008).
13. Government of India, **29th Report of the Standing Committee on Urban Development** (Ministry of Urban Development ed., 2008).
14. Government of India, **The Unorganised Workers' Social Security Act, 2008** (Ministry of Labour and Employment ed., 2008).
15. Government of India, **Report of the Working Group on Labour Laws and Other Labour Regulations for the 12th Five Year Plan (2007-12)** (Ministry of Labour and Employment ed., 2011).
16. Government of **Karnataka, Karnataka Mental Health Rules, 2012** (Department of Health and Family Welfare ed., 2012).
17. Government of India, **National Urban Health Mission: Framework for Implementation** (Ministry of Health and Family Welfare ed., 2013).
18. Government of India, **The National Food Security Act, 2013** (Ministry of Law and Justice ed., 2013).
19. Government of India, **The Prohibition of Employment as Manual Scavengers and their Rehabilitation Act, 2013** (Ministry of Social Justice and Welfare ed., 2013).
20. Government of India, **The Varishtha Pension Bima Yojana, 2014** (Ministry of Finance ed., 2014).
21. Government of India, **Pradhan Mantri Jeevan Jyoti Bima Yojana (PMJJBY)** (Ministry of Finance ed., 2015).
22. Government of India, **The Solid Waste Management Rules, 2016** (Forest and Climate Change Ministry of Environment ed., 2016).
23. Government of India, **Pradhan Mantri Matru Vandana Yojana (PMMVY), 2017: Scheme Implementation Guidelines** (Ministry of Health and Family Welfare ed., 2017).
24. Government of India, **The Mental Healthcare Act, 2017** (Ministry of Law and Justice ed., 2017).

25. Government of India, **Ayushman Bharat – Pradhan Mantri Jan Arogya Yojana (PM-JAY): Policy & Guidelines** (Ministry of Health and Family Welfare ed., 2018).
26. Government of **Himachal Pradesh, Himachal Pradesh Mukhya Mantri Chikitsa Sahayata Kosh, 2018** (Department of Health and Family Welfare ed., 2018).
27. Government of India, **A Report of High Level Group on Health Sector: Submitted to Fifteenth Finance Commission** (Finance Commission of India ed., 2019).
28. Government of Maharashtra, **Integrated Mahatma Jyotirao Phule Jan Arogya Yojana (MJPJAY) and Ayushman Bharat-Pradhan Mantri Jan Arogya Yojana (AB-PMJAY), 2020** (Department of Health and Family Welfare ed., 2020).
29. Government of India, **Third Report of the Standing Committee on Urban Development (2019-2020)** (Ministry of Housing and Urban Affairs ed., 2020).
30. Government of India, **The Code on Social Security, 2020** (Ministry of Law and Justice ed., 2020).
31. Government of India, **The Occupational Safety, Health and Working Conditions Code, 2020** (Ministry of Law and Justice ed., 2020).
